# Integrin α4β1 controls G9a activity that regulates epigenetic changes and nuclear properties required for lymphocyte migration

**DOI:** 10.1093/nar/gkv1348

**Published:** 2015-12-10

**Authors:** Xiaohong Zhang, Peter C. Cook, Egor Zindy, Craig J. Williams, Thomas A. Jowitt, Charles H. Streuli, Andrew S. MacDonald, Javier Redondo-Muñoz

**Affiliations:** 1 Wellcome Trust Centre for Cell Matrix Research, Faculty of Life Sciences, University of Manchester, Manchester, M13 9PT, UK; 2 Manchester Collaborative Centre for Inflammation Research, University of Manchester, Manchester, M13 9NT, UK; 3 Materials Science Centre, School of Materials, University of Manchester, Manchester, M13 9PL, UK

## Abstract

The mechanical properties of the cell nucleus change to allow cells to migrate, but how chromatin modifications contribute to nuclear deformability has not been defined. Here, we demonstrate that a major factor in this process involves epigenetic changes that underpin nuclear structure. We investigated the link between cell adhesion and epigenetic changes in T-cells, and demonstrate that T-cell adhesion to VCAM1 *via* α4β1 integrin drives histone H3 methylation (H3K9me2/3) through the methyltransferase G9a. In this process, active G9a is recruited to the nuclear envelope and interacts with lamin B1 during T-cell adhesion through α4β1 integrin. G9a activity not only reorganises the chromatin structure in T-cells, but also affects the stiffness and viscoelastic properties of the nucleus. Moreover, we further demonstrated that these epigenetic changes were linked to lymphocyte movement, as depletion or inhibition of G9a blocks T-cell migration in both 2D and 3D environments. Thus, our results identify a novel mechanism in T-cells by which α4β1 integrin signaling drives specific chromatin modifications, which alter the physical properties of the nucleus and thereby enable T-cell migration.

## INTRODUCTION

Cell migration is critical for numerous biological processes, including embryogenesis, tissue repair and immune responses (1,2). Current concepts suggest that cells when migrating are highly deformable and this is necessary in order to migrate through narrow tissue spaces (3). Indeed, it is implied that for effective cell migration, the nucleus, which is the major and most intrinsically rigid organelle in the cell, must alter its mechanical properties (4). Important structural changes in the nucleus occur through epigenetics, which involve chromatin changes that modulate gene expression. Chromatin can be configured as euchromatin, in which it has an open conformation and it is then associated with active transcription, whereas as heterochromatin it is condensed and forms an inactive configuration (5). These epigenetic changes involve specific histone variants and DNA and histone modifications, which affect the chromatin structure in response to biological signals (6). One important epigenetic change is the methylation of lysine 9 in histone H3, which is mediated by several histone methyltransferases (HMT's), including G9a, G9a-like protein (GLP), PR domain zinc finger protein 2 (PRDM2), SUVH1/2 and SETDB1/ESET (7–9). Moreover, this histone lysine methylation, as well as other epigenetic methylations such as H4K20me3, has been correlated with active cell migration (9,10). However, the mechanisms connecting these changes in the nucleus with cell migration are unclear.

Lymphocytes, B- and T-cells, are immune cells involved in adaptive immunity. Amongst T-cell sub-types are CD8^+^ cells involved with cytotoxic responses, whilst CD4^+^ cells are active in cytokine production, regulatory functions and tolerance responses. Under different stimuli, T-cells migrate rapidly through tissue barriers, such as endothelium and also through the dense extracellular matrix (ECM) of different tissues (11). Integrins control lymphocyte adhesion to endothelial cells and govern their extravasation into inflamed tissues (12–14). The integrin α4β1 (CD49d/CD29), which binds VCAM1 (Vascular Cell Adhesion Molecule-1) and fibronectin, is critical for lymphocyte adhesion, extravasation and activation (15). Aberrant expression and altered function of α4β1 has been described in multiple autoimmune diseases and in cancer (16,17). Understanding the mechanisms that connect cell adhesion and epigenetic changes with lymphocyte migration could identify new therapeutic targets for inflammatory and immune disorders.

Here, we investigated how lymphocyte adhesion through α4β1 integrin induced global epigenetic changes in H3K9me2/3 levels, which correlated with changes in the physical properties of the T-cell nucleus. We identified G9a as the enzyme responsible for these epigenetic changes and showed how this affected T-cell migration. Together, our results reveal a novel mechanism linking cell adhesion through integrins to govern chromatin changes in the nucleus and thereby modify the physical properties of the nucleus to enable efficient T-cell migration.

## MATERIALS AND METHODS

### Cells

The human T-cell line Jurkat was obtained from Dr Christoph Ballestrem (University of Manchester, UK). For primary T-cell isolation, CD4+ T cells were positively selected from spleen and LN of C57BL/6 mice, using CD4+ microbeads (Miltenyi Biotec; Bergisch Gladbach, Germany) following the manufacturers protocol. Mice on a C57BL/6 background were maintained in the Faculty of Life Sciences, University of Manchester, in compliance with the UK Home Office Animals (Scientific Procedures) Act 1986. Primary T-cells and Jurkat were maintained in RPMI 1640 medium (Gibco) with HEPES (10 mM), L-glutamine (2 mM), 10% fetal calf serum and 1% penicillin/streptomycin, in 5% CO2 at 37°C. Human HEK293T cells were cultured in DMEM (Gibco), L-glutamine (2 mM), supplemented with 10% fetal calf serum and 1% penicillin/streptomycin. All cells were cultured in 5% CO2 at 37°C.

### Reagents and antibodies

The mouse antibody anti-H3K9me2/3, and the rabbit antibodies anti-H3K9ac, -H3K4me3, -H4K20me3, -H3 and -G9a were from Cell Signaling (Beverly, MA, USA). Rabbit anti-GLP was from Thermo- Scientific (Waltham, MA, USA). Mouse anti-lamin B1 (for the HMT experiment) was from Santa Cruz Biotechnology (Dallas, TX, USA) and rabbit anti-lamin B1 was from Abcam (Cambridge, UK). The rabbit antibody against suv39h1 was from Abcam and the mouse anti-β-tubulin was from Sigma-Aldrich (St. Louis, MO, USA). Anti-CD3 and anti-CD28 were from Biolegends (San Diego, CA, USA). 12G10 (anti-β1 activator Ab), 9EG7 (anti-β1 activator Ab) and mab13 (anti-β1 blocking Ab) were kindly provided by Martin Humphries (University of Manchester, UK). VCAM1 was obtained from Martin Humphries and Peprotech. Fibronectin fragment FN-H50 and FN-H120 were kindly provided by Martin Humphries. Fluorescence-conjugated secondary antibodies were from Jackson ImmunoResearch (West Grove, PA, USA). ICAM1, IL-4, CXCL12 and CCL19 were from Peprotech (Rocky Hill, NJ, USA). BIX01294, chaetocin, actinomycin were from Sigma-Aldrich. Hoechst 33342 was from Invitrogen (Grand Island, NY, USA). CFSE and Cell Tracer Far Red were from Life Technologies (Paisley, UK).

### AbFRET (acceptor photobleaching fluorescence resonance energy transfer)

Jurkat cells were electroporated with pcDNA3-K9 histone methylation reporter, which was a gift from Alice Ting (18; Addgene plasmid # 22866). Briefly, 5 × 10^6^ cells were incubated in 400 μl of OPTIMEM (Gibco) medium in the presence of 10 μg of DNA for 10 min on ice. Then, cells were electroporated in a Gene Pulse II (BioRad; Hercules, CA) at 250 V, 975 μF and kept on ice for an additional 5 min. Then, cells were diluted in complete medium and cultured for 24 h prior the experiment. Cells were cultured onto glass-bottomed well plate (Mattek; Ashland, MA, USA) coated with poly-Lysine or VCAM1, in the presence or absence of different inhibitors. FRET efficiency was examined by AbFRET method on an inverted confocal (TCS SP5 AOBS; Leica) with 63x, 1.4 NA HCX Plan Apochromat oil objectives and LAS AF acquisition software (Leica). A region of interest (ROI) was drawn over the selected region and 50% at 514 nm for 8 iterations was used for photobleaching the acceptor signal. Prebleach and Postbleach images were acquired using identical imaging settings. The difference of donor intensities before (D_pre_) and after (D_post_) acceptor photobleaching gives the FRET efficiency as follows:

\begin{equation*} {\rm FRET}_{{\rm eff}} = ({\rm D}_{{\rm post}} - {\rm D}_{{\rm pre}} )/{\rm D}_{{\rm post}} \end{equation*}

### HMT activity

G9a activity was measured in nuclear extracts or IP fractions of Jurkat cells cultured in suspension or onto VCAM1 according to the manufacturer's instructions (EpiQuik Histone Methyltransferase Activity/Inhibition Assay Kit; Epigentek, Farmingdale, NY, USA). HMT activity was detected by using a microplate reader at 450 nm.

### DNaseI-sensitivity assay

For *in situ* DNAseI sensitivity assay we follow a previous protocol (19). Briefly, Jurkat cells were cultured on different ligands and lysed in CSK buffer for 5 min and then DNAseI (New England Biolabs; Ipswich, MA, USA) was incorporated for 20 min. The remaining DNA was washed once with CSK buffer plus 125 mM ammonium sulfate and then stained by Hoechst 33342 in the same buffer for 5 min at RT. Nuclei were fixed in methanol at −20°C for 5 min, and coverslips washed in CSK buffer, mounted using Dako (H-1000, Vector Laboratories; Peterborough, UK), and analysed by confocal microscopy.

### Atomic Force Microscopy (AFM)

Nuclei from cells cultured in suspension, on ICAM1 or VCAM1 (with and without inhibitors) were isolated after subcellular fractionation (20), and sedimented onto poly-Lysine coated slides (Thermo Scientific). Then, nuclei were fixed with PBS (Phosphate buffered saline) 2% paraformaldehyde for 10 min, washed with PBS and water, and let dried until their analysis. AFM was performed on a Bruker (Coventry, UK) Catalyst head mounted to a Nikon inverted Microscope. Experiments were performed using a Bruker Scan Assist Fluid Probe. Probes were calibrated using Bruker NanoScope software on a bare glass slide. Samples were imaged in double distilled water using Scan Assist mode, typically at 64 × 64 pixels over 20–40 μm scan sizes. For mechanical testing, 20 nuclei per sample were tested at 9 indents per nuclei, using a ramp size of 500 nm and a trigger threshold of 25 nm. The force curves were analysed using Bruker NanoScope Analysis. The approach curve was fitted using a contact point based Sneddon model. From this, the reduced modulus of each point was taken and averaged for each nucleus.

### Statistical analysis

Statistical analysis and comparisons were made with GraphPad Prism6 (GraphPad Software, La Jolla, CA, USA). Differences between means were tested by Student's *t-*test for normal data. Where three or more groups were analysed, one-way ANOVA was used. *P*-values are indicated by asterisks ((*)*P* < 0.05; (**)*P* < 0.01; (***)*P* < 0.001).

## RESULTS

### T-cell adhesion *via* α4β1 integrin increases H3K9 methylation in the nucleus

**Figure 1.**
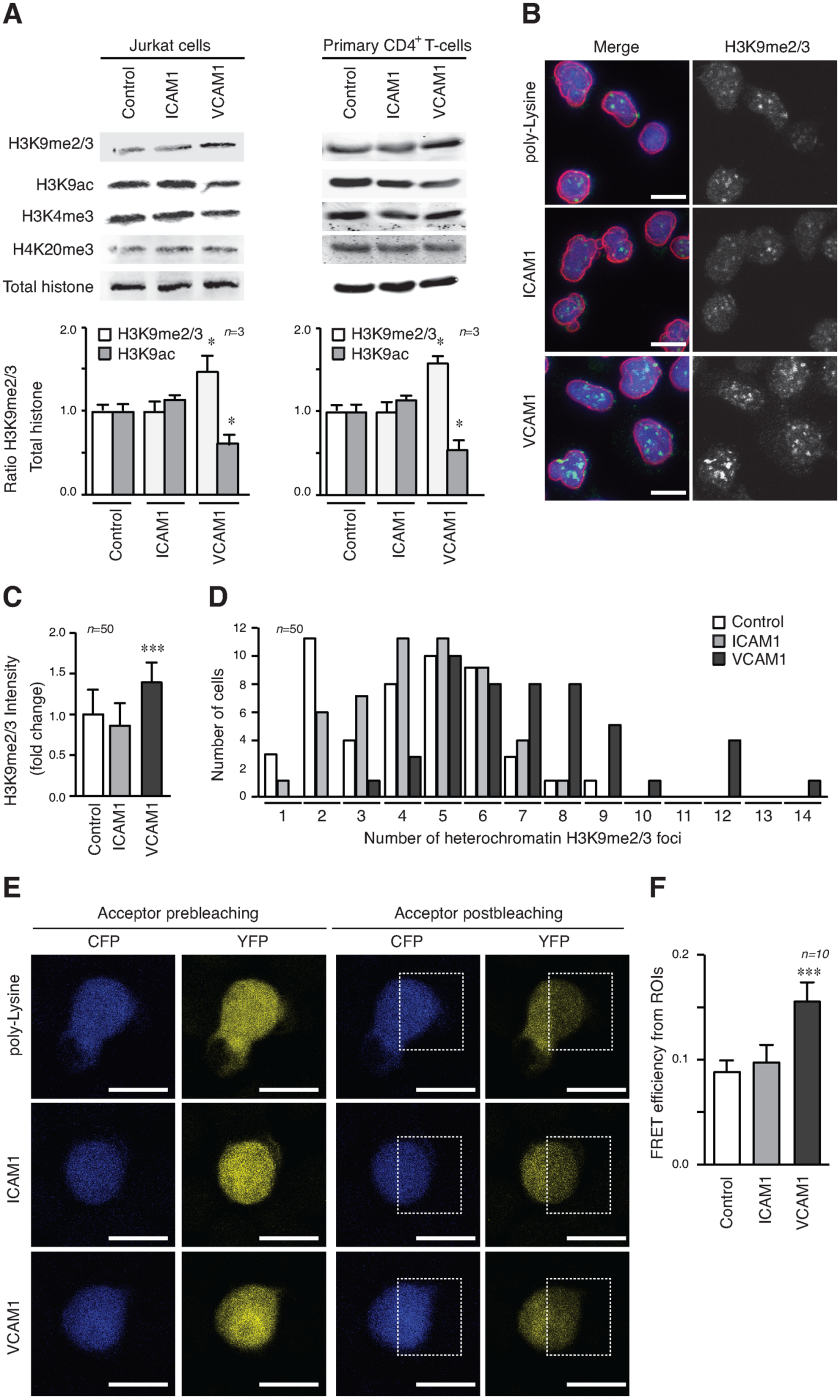
T-cell adhesion to VCAM1 induces H3K9 methylation. (**A**) Jurkat (left panel) or primary CD4^+^ T-cells (right panel) were cultured in suspension (Control), on ICAM1 (5 μg/ml) and VCAM1 (5 μg/ml) for 24 h and lysates analysed by Western blotting. Graphs show the ratio of H3K9me2/3 versus total histone and normalised respect to control cells. (**B**) Jurkat cells cultured on poly-Lysine, ICAM1 or VCAM1 (all at 5 μg/ml) were fixed and stained with Hoechst (DNA, blue), anti-lamin B1 (nuclear envelope, red) and anti-H3K9me2/3 (heterochromatin, green). Bar 10 μm. (**C**) Graph shows the quantification of H3K9me2/3 fluorescence intensity of cells in (B). (**D**) Quantification of the increase of heterochromatin foci, as seen by number of H3K9me2/3 foci counted per nucleus in (B). (**E**) Jurkat cells transfected with a FRET-H3K9 methylation biosensor and were cultured on poly-Lysine, ICAM1 or VCAM1. Images show the CFP and YFP signals before and after YFP photobleaching at 514 nm in the area delineated (ROI, region of interest). Bar 10 μm. (**F**) Graph represents the quantification of FRET efficiency from the pre- and post-donor fluorescence intensities in the photobleached area (ROI). The average FRET efficiency is expressed as the mean ± S.E. From 10 cells. *P < 0.05; **P < 0.01; ***P < 0.001.

To explore the role of integrins in epigenetic modifications, we cultured Jurkat on VCAM1, and ICAM1 (intercellular adhesion molecule-1), which are ligands for integrins α4β1 and αLβ2 (CD11a/CD18), respectively. Adhesion *via* α4β1 caused an increase in H3K9me2/3, which is a heterochromatin marker strongly associated with cell migration, compared to cells in suspension, or cells cultured on ICAM1 (Figure 1A). Primary CD4^+^ T-cells also showed H3K9me2/3 upregulation upon VCAM1 adhesion, although this change was more moderate than Jurkat cells. This increase in H3K9me2/3 correlated with a decrease in the euchromatin marker H3K9ac, without any significant effect on other heterochromatin (H4K20me3) or euchromatin (H3K4me3) markers (Figure 1A).

To confirm these effects of T-cell adhesion to VCAM1 on cell chromatin, we performed immunofluorescence localisation of H3K9me2/3 in Jurkat cells plated on different substrates (Figure 1B). Quantification of the fluorescence intensity for each nucleus showed that the total H3K9me2/3 was significantly higher in Jurkat cells plated on VCAM1 compared with those plated on poly-Lysine or ICAM1 (Figure 1C). We also determined the number of H3K9me2/3 foci in the different conditions and showed a marked increase when Jurkat cells were plated on VCAM1 compared to the other conditions (Figure 1D), whilst H4K20me3 localization remained unaltered (Supplementary Figure S1). To further substantiate the quantitative increase in H3K9me2/3, we used a CFP-YFP H3K9 biosensor that recognises H3K9 methylation (me2 and me3) to measure levels of H3K9 methylation by FRET efficiency (21). The validity of this assay was confirmed by photobleaching the acceptor (YFP) in a region of the cells and quantifying the subsequent increase of the donor (CFP). Jurkat cells plated on poly-Lysine or ICAM1 gave a lower FRET efficiency, which corresponds to H3K9 methylated levels, compared to those plated on VCAM1 (Figure 1E and F), indicating that the probe recognised higher levels of H3K9 methylation in the Jurkat cells on VCAM1 consistent with our initial results.

Together, these results provided strong evidence that epigenetic changes in T lymphocytes were driven by cell adhesion to VCAM1.

### Interaction of α4β1 integrin with VCAM1 triggers the epigenetic changes induced by T-cell adhesion.

**Figure 2.**
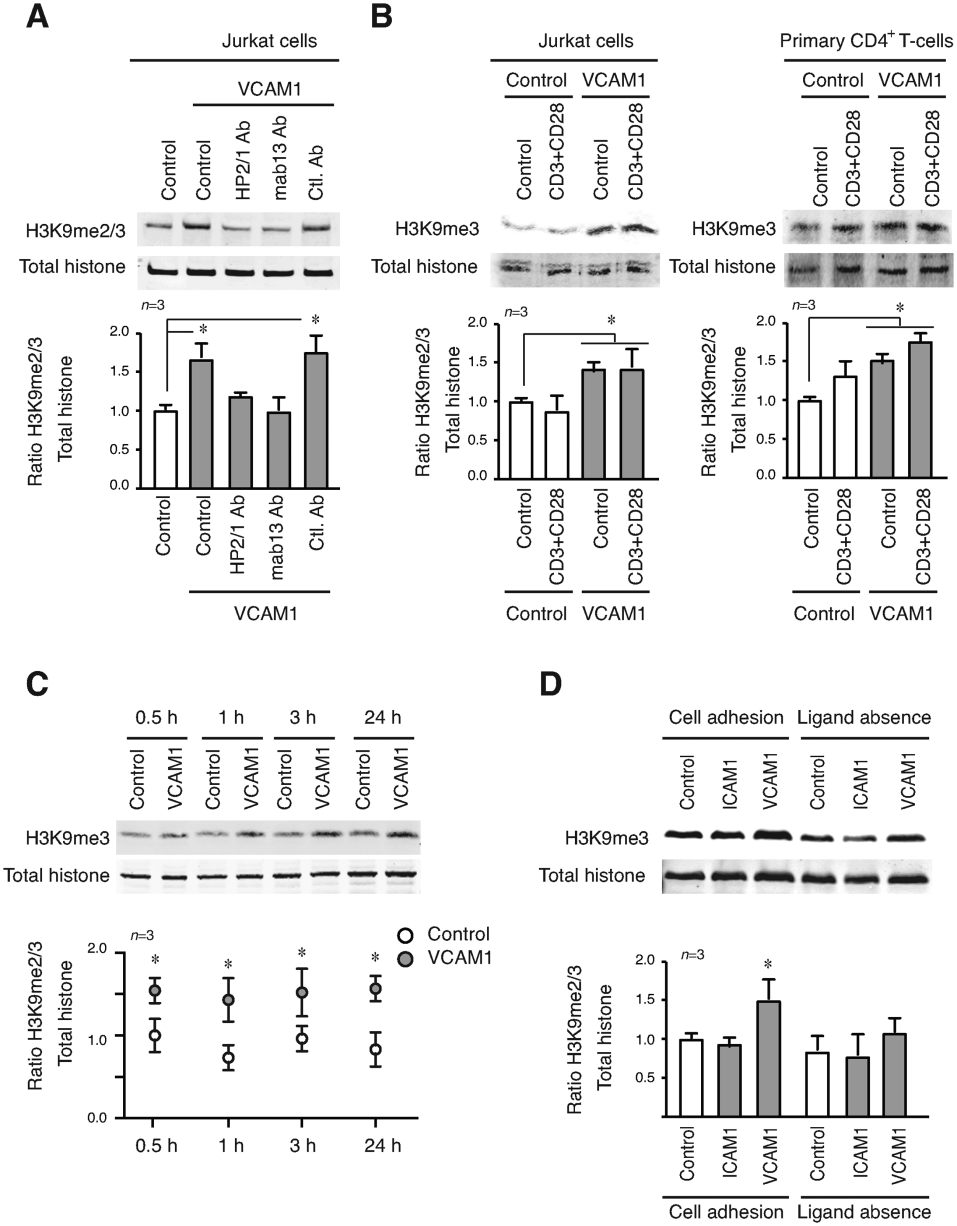
α4β1 integrin-VCAM1 interaction is essential for the epigenetic changes induced upon T-cell adhesion. (**A**) Jurkat were preincubated with blocking antibodies against -α4 (HP2/1) and -β1 (mab13) integrin subunits; or a control antibody (all of them at 10 μg/ml), 30 min prior cell culture onto VCAM1. Cells were lysed after 24 h and protein expression analysed by Western blotting. (**B**) Jurkat (left panel) or primary CD4^+^ T-cells (right panel) were cultured for 24 h in the presence of anti-CD3 and anti-CD28 antibodies. Epigenetic changes were analysed by Western blotting. (**C**) Jurkat cells were cultured in suspension or on VCAM1 at different times, lysed and H3K9me2/3 levels quantified by western blotting. (**D**) Jurkat cells were cultured on different conditions for 24 h, collected and cultured for an additional 24 h in the absence of any ligand. Lysates were analysed by Western blotting and quantified. *P < 0.05.

To verify that α4β1 was the specific cell receptor involved in the histone methylation induced by adhesion to VCAM1, we treated Jurkat cells (Figure 2A) or primary CD4^+^ T-cells (Supplementary Figure S2A) with integrin-blocking antibodies prior to VCAM1 adhesion. We showed that antibodies against the -α4 (HP2/1) and -β1 (mab13) subunits completely blocked H3K9 methylation induced by cell adhesion to VCAM1. We observed, using recombinant fragments of fibronectin (22), that Jurkat cells adhesion *via* α4β1 to FN-H120 (ligand for α4β1) caused an increase in H3K9me2/3 similar to that seen after adhesion to VCAM1. In contrast, FN-H50 (ligand for α5β1) did not affect H3K9me2/3 levels. Primary CD4^+^ T-cells showed similar changes, although they were more modest than those in Jurkat cells (Supplementary Figure S2B).

Integrins are receptors that cycle between inactive and active forms, depending on different (outside-in or inside-out) signals (12), we assessed whether activation of the integrin was enough to trigger the epigenetic changes. We determined that divalent cations or activator anti-β1 mAbs had no significant effect on H3K9me2/3 levels in Jurkat cells (Supplementary Figure S2C). We also incubated Jurkat cells, or primary CD4^+^ T-cells with anti-CD3 and anti-CD28 antibodies (to induce T-cell receptor stimulation) in the presence of VCAM1, but did not see any significant variation in H3K9me2/3 levels (Figure 2B).

The pattern of histone methylation generated during migration of invasive tumour cells is reported to persist well beyond the phase of migration (10). We therefore analysed the time course of H3K9me2/3 methylation generated during and beyond T-cell adhesion through α4β1. We observed that 30 min after culturing Jurkat cells on VCAM1 the level of H3K9me3 increased and remained unaltered for at least 24 h (Figure 2C). However, H3K9me2/3 induced by cell adhesion to VCAM1 was lost if the cell culture was continued in the absence of the integrin ligand for 24 h (Figure 2D).

These results showed clearly that blocking α4β1 integrin affects the epigenetic changes induced by cell adhesion to VCAM1 and that the integrin–ligand interaction is necessary to sustain these changes.

### α4β1 integrin adhesion controls H3K9 methylation through G9a histone methyltransferase

**Figure 3.**
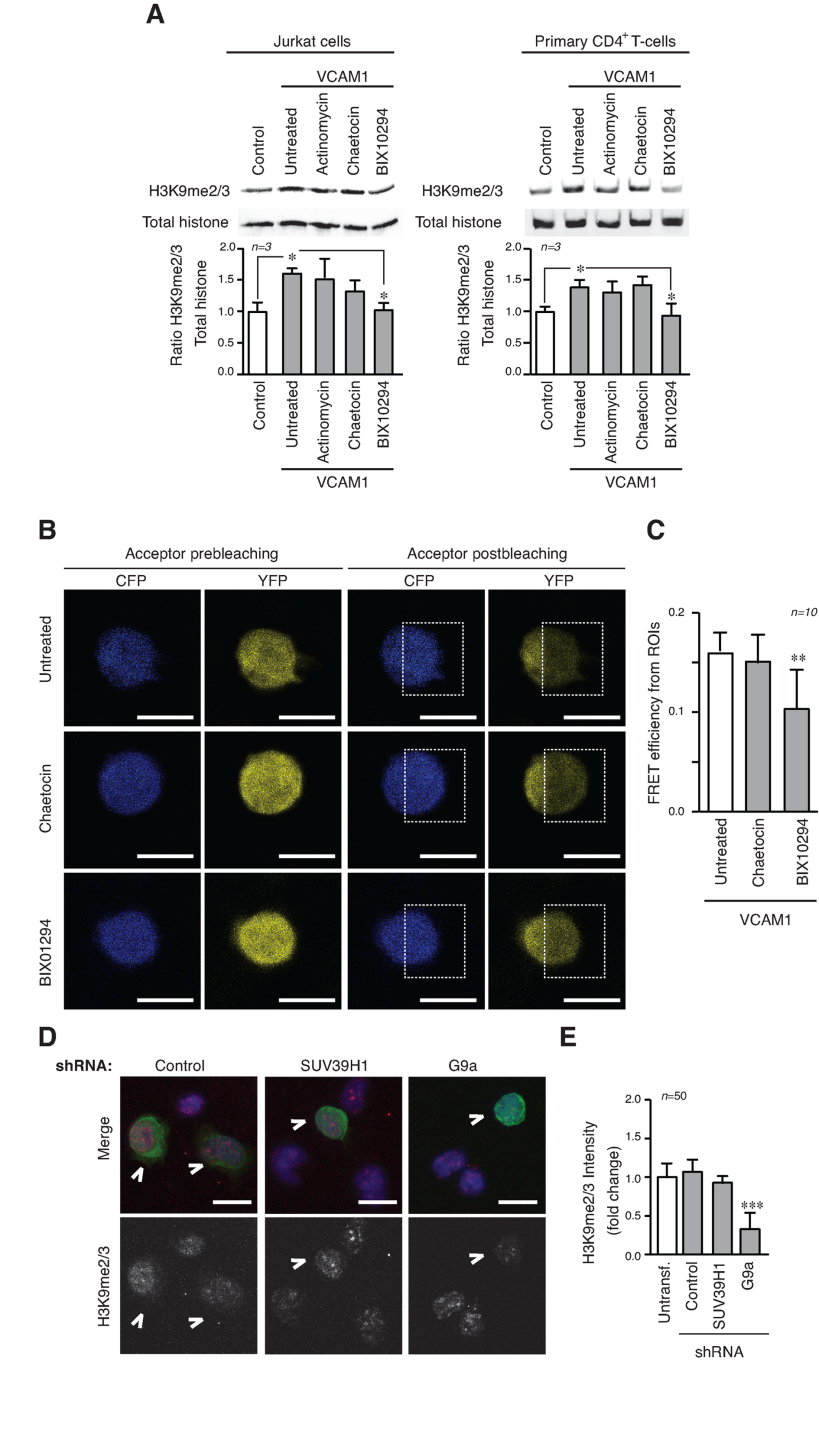
The activity of the HMT G9a is required for the epigenetic changes induced by α4β1 adhesion. (**A**) Jurkat (left panel) or primary CD4^+^ T-cells (right panel) were cultured on VCAM1 in the presence of actinomycin (transcriptional inhibitor), chaetocin (Suv39H inhibitor) or BIX01294 (G9a/GLP inhibitor). After 24 h, cells were lysed and proteins levels analysed by Western blotting. (**B**) Jurkat cells transfected with the H3K9 FRET methylation biosensor were cultured as in (A). CFP and YFP signals were measured pre- (left panels) and post-YFP photobleaching (right panels) at 514 nm in the area delineated. Bar 10 μm. (**C**) Graph represents the FRET efficiency mean calculated from the pre- and post-donor fluorescence intensities in the photobleached area. (**D**) Untransfected or stable GFP^+^ shRNA for G9a, SUV39H1 and control Jurkat cells were mixed and cultured on VCAM1. After 24 h, cells were fixed and stained with Hoechst (blue) and anti-H3K9me2/3 (red). The arrows mark stable transfected cells. Bar 10 μm. (**E**) Graph shows the quantification of H3K9me2/3 fluorescence intensity of cells in (D). *P < 0.05; **P < 0.01; ***P < 0.001.

To identify which HMT transmitted the signals induced by α4β1, we used specific HMT inhibitors (chaetocin for SUV39H1 and BIX01294 for G9a/GLP) or actinomycin to inhibit transcription to rule out that defective transcription was responsible for this effect. Only BIX01294 inhibitor blocked the H3K9me2/3 upregulation mediated in Jurkat cells or primary CD4^+^ T-cells on VCAM1 (Figure 3A and Supplementary Figure S3A) and showed lower levels of FRET efficiency in transfected cells FRET-H3K9 biosensor than untreated or chaetocin treated cells (Figure 3B and C). We also verified that Jurkat cells were able to adhere efficiently to ICAM1 and VCAM1 in the presence of HMT inhibitors, showing that the results were not due to altered cell adhesion (Supplementary Figure S3B). Using lentiviral shRNA we generated stable cell lines depleted of SUV39H1, or G9a (Supplementary Figure S3C). Experiments with these cells plated on VCAM1 showed that in G9a-depleted cells there were fewer H3K9me2/3 foci of weaker intensity compared with untransfected, control and SUV39H1-depleted cells (Figure 3D and E). We confirmed that depletion of G9a reduced the levels of H3K9me2/3 when cells were cultured on VCAM1 (Supplementary Figure S3D).

Together, these results confirmed the central role of G9a in mediating the epigenetic changes induced by α4β1 during cell adhesion.

### T-cell adhesion through α4β1 regulates G9a localization and activity

Integrin α4β1 signaling controls the Th1/Th2 balance in T-cells (23). As G9a is reported to play a critical role in the biology of CD4^+^ T-cells during Th2 differentiation in immune responses (24,25), we assessed whether cell adhesion to VCAM1 might promote epigenetic changes *via* Th2 response. Interleukin-4 (IL-4), which is a well-known inductor of the Th2 response, did not alter H3K9me2/3 levels in Jurkat cells plated on VCAM1 (Supplementary Figure S4A). Furthermore, the expression of Th2 cytokines were not affected when Jurkat cells were in suspension, or plated on ICAM1 or VCAM1 (Supplementary Figure S4B), indicating that the epigenetic changes induced by α4β1 are independent of the changes driving cytokine production, or the Th2 response.

**Figure 4.**
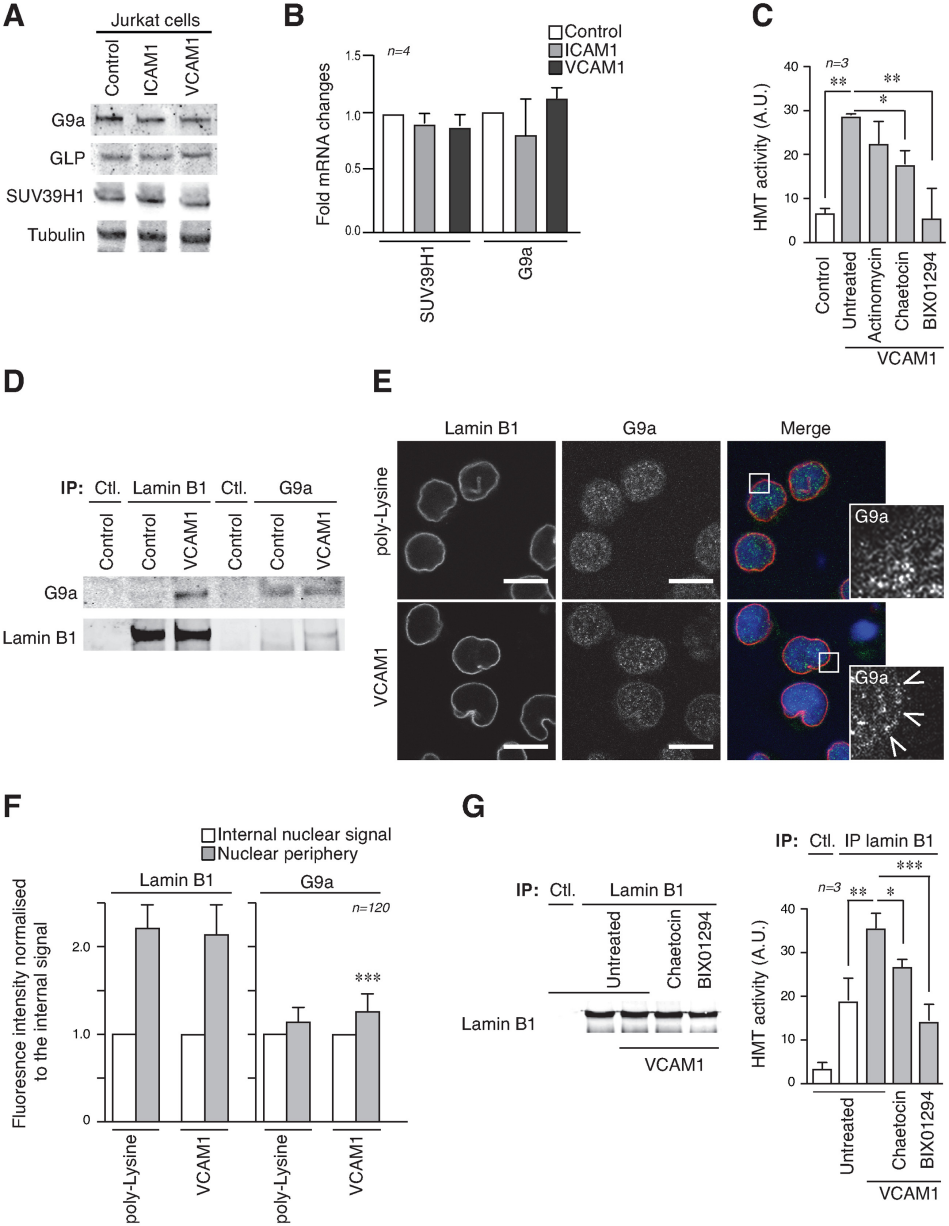
Cell adhesion controls G9a activity and its interaction with the nuclear envelope. (**A**) Jurkat cells were cultured on different substrates and protein expression analysed by Western blotting. (**B**) RNA was isolated from Jurkat cells as in (A). RT-qPCR analysis was performed using primers directed at specific genes, as indicated. Values were calculated as the mean relative expression after normalization to two housekeeping genes 6 SEM. (**C**) *In vitro* H3K9 HMT enzymatic assay from nuclear fractions of Jurkat cells cultured in suspension or on VCAM1 in the presence or not of different inhibitors. (**D**) Complexes (IP) were precipitated from Jurkat cells cultured in suspension or on VCAM1 for 24 h with anti-lamin B1 and anti-G9a antibodies and probed with different antibodies. (**E**) Jurkat cells were cultured on poly-Lysine or VCAM1 and then fixed and stained with Hoechst (blue), anti-lamin B1 (red) and anti-G9a (green). Arrows indicate G9a localisation at the nuclear envelope. Bar 10 μm. (**F**) Lamin B1 and G9a signals were quantified at the nuclear periphery or inside the nucleus. (**G**) Lamin B1 fractions immunoprecipitated from Jurkat cells cultured under different conditions were resolved by Western blotting. Graph shows the HMT activity associated to lamin B1 immune complexes. *P < 0.05; **P < 0.01; ***P < 0.001.

Protein and mRNA analysis showed that G9a or SUV39H1 were not upregulated when Jurkat cells were cultured on VCAM1 (Figure 4A and B), however, we found higher levels of HMT activity in the nuclear fractions of Jurkat cells cultured on VCAM1 compared to control cells, or cells treated with BIX01294 (Figure 4C).

As chromatin and HMTs are associated with nuclear lamins (26–28), we investigated the localisation of G9a following cell adhesion though α4β1. We determined that anti-lamin B1 antibodies pulled down G9a, but not SUV39H1, from lysates of Jurkat cells when these were cultured on VCAM1, but not when they were in suspension (Figure 4D). To confirm this we determined G9a localisation by immunofluorescence, using lamin B1 staining as a reference for the nuclear periphery (Figure 4E). This showed that G9a localisation was greatest at the nuclear envelope compared to inside the nucleus when Jurkat cells were plated on VCAM1 (Figure 4F). Moreover, we detected higher HMT activity in the lamin B1-associated fraction from Jurkat cells plated on VCAM1 compared to cells in suspension, or treated with BIX01294 inhibitor (Figure 4G). This confirmed that α4β1 adhesion induces HMT activity associated at the nuclear envelope. To further validate this result, we showed that lamin B1-depleted Jurkat cells had defective H3K9 methylation induced by α4β1 in their nuclear fractions (Supplementary Figure S5).

These results demonstrated that α4β1 integrin controls G9a activity and its interaction with lamin B1. This identified a connection between chromatin methylation and structural elements of the nuclear envelope, which may contribute to the changes in the physical properties of the nucleus.

### Epigenetic changes induced by α4β1 control both chromatin conformation and physical properties of the nucleus

**Figure 5.**
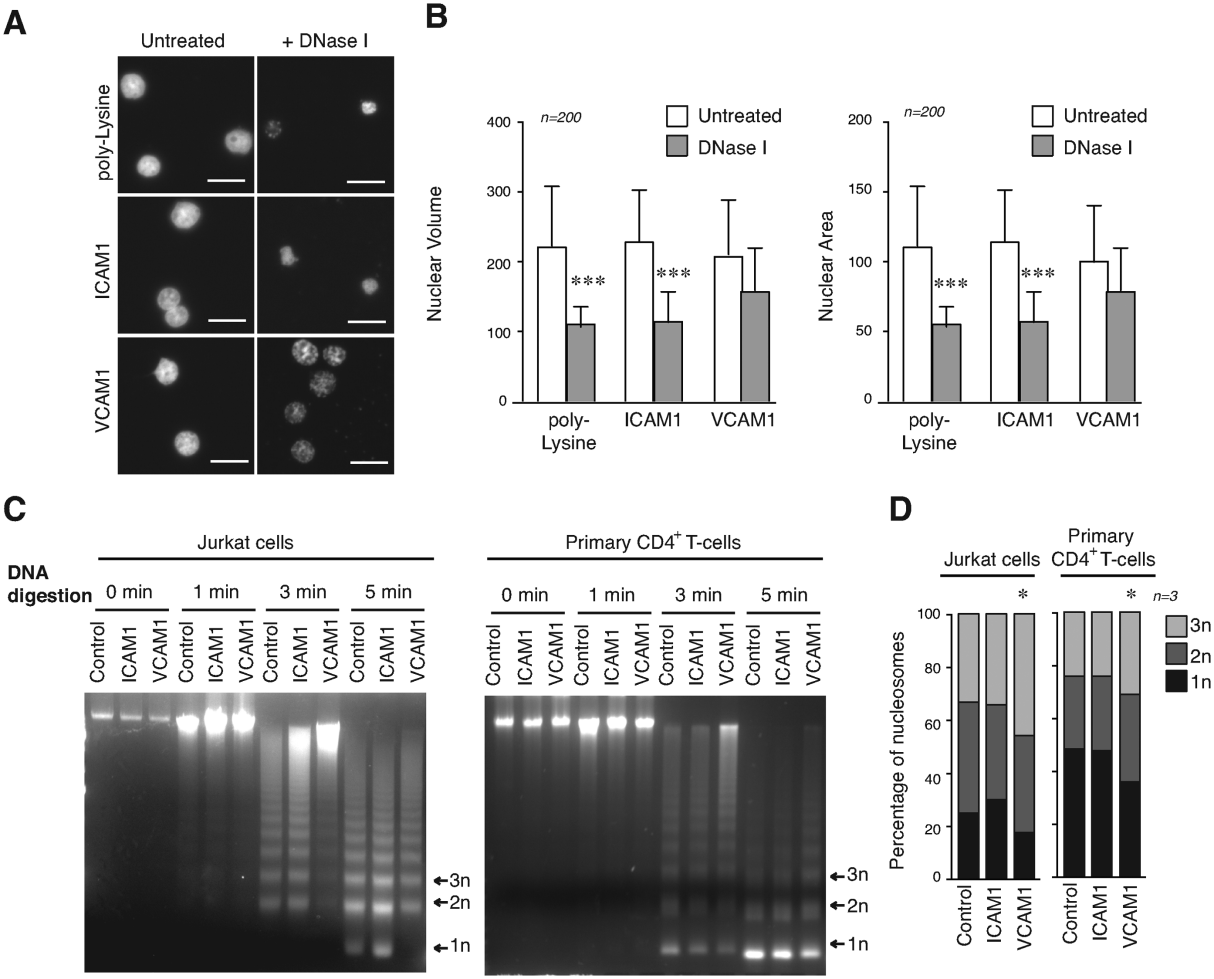
Cell adhesion through α4β1 regulates global chromatin conformation. (**A**) Jurkat cells were cultured on poly-Lysine, ICAM1 or VCAM1 for 24 h. Cytoskeleton was removed by extensive CSK buffer washes and DNA was digested by DNaseI before fixation. Remaining DNA was stained with Hoechst. Bar 10 μm. (**B**) Graphs show the quantification of the nuclear volume and area from cells in (A). (**C**) Jurkat (left panel) or primary CD4^+^ T-cells (right panel) were cultured in suspension, on ICAM1 and VCAM1 for 24 h. Then, cells were digested with micrococcal nuclease at indicated times. DNA fragments were purified and resolved in agarose gel. (**D**) Nucleosomal releasing from cells in (C) was analysed after micrococcal digestion and the mononucleosomes (1n), dinuclueosomes (2n) and trinucleosomes (3n) quantified. *P < 0.05; ***P < 0.001.

We studied how G9a activity and increased levels of H3K9me2/3 alter chromatin structure in T-cells. We determined that the volume and area of the nucleus in cells cultured on poly-Lysine or ICAM1 were smaller after DNA digestion than nuclei of cells on VCAM1, which suggested that then nuclei from cells on VCAM1 were more resistant to DNA digestion (Figure 5A and B). Moreover, cells treated with BIX01294 and cultured on VCAM1 were more also sensitive to digestion (Supplementary Figure S6A). We determined nucleosome release after microccocal enzyme (MNase) digestion (Figure 5C). Nucleosomes are the basic unit of chromatin and linked to chromatin compaction (29). Whilst Jurkat in control conditions, or cultured on ICAM1, showed a high release of mononucleosomes after 5 min of MNase digestion, cells on VCAM1 were much more resistant to digestion. A moderate, yet significant effect was also observed using primary CD4^+^ T-cells cultured on VCAM1 (Figure 5D). BIX01294 treatment reversed this resistance to DNA digestion induced by adhesion to VCAM1, while only a partial effect was observed with chaetocin and no effect with actinomycin (Supplementary Figure S6B). These results provided evidence that α4β1 adhesion induces a more closed conformation of the chromatin.

**Figure 6.**
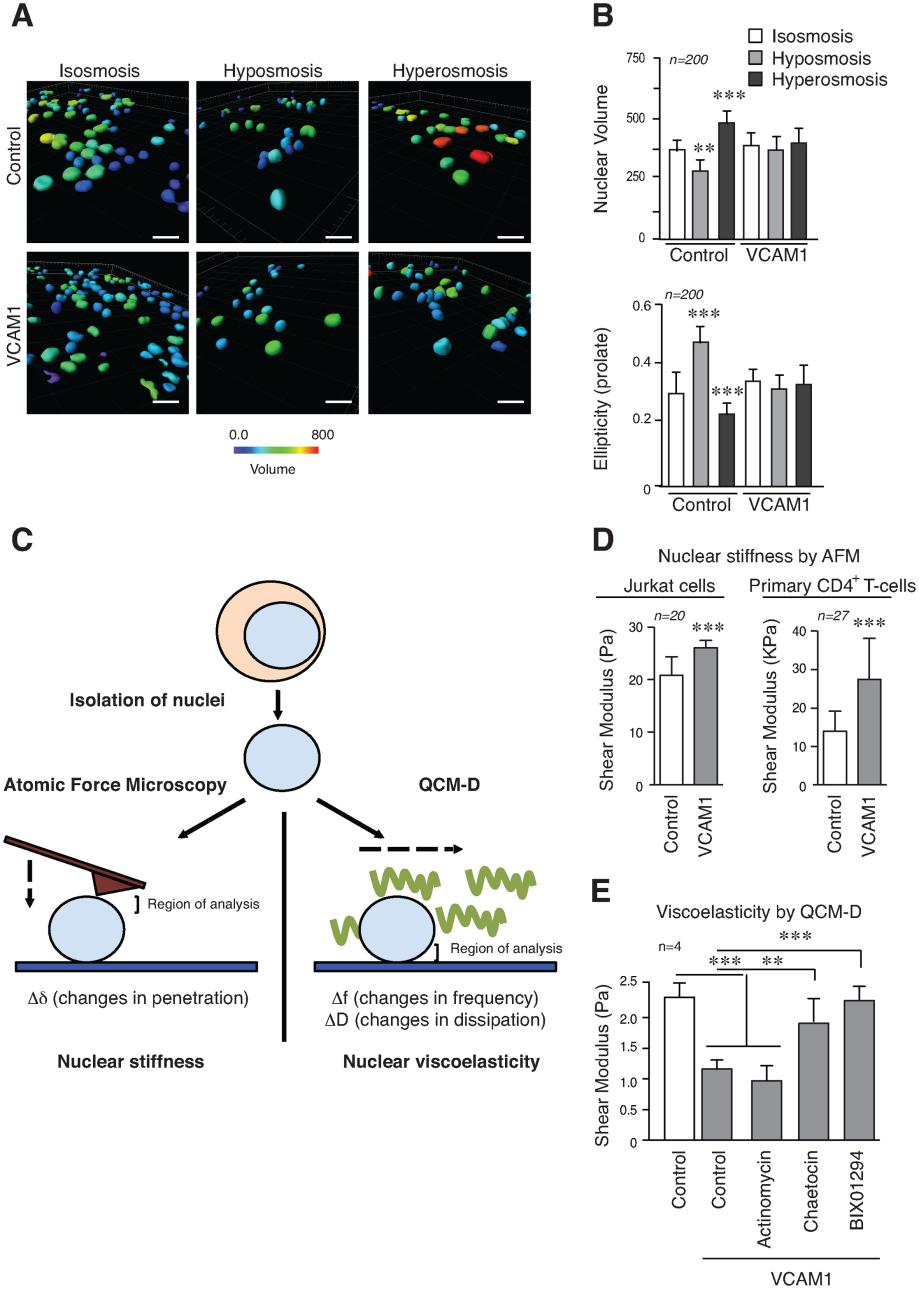
Epigenetic changes induced by α4β1 contribute to nuclear stiffness and viscoelasticity. (**A**) Jurkat cells were cultured on poly-Lysine or VCAM1 for 24 h and then medium was changed for isotonic, hypotonic or hypertonic medium to induced osmotic stress. Cells were fixed, stained with Hoechst and analysed by confocal microscopy. Reconstruction of nuclei was done from images and changes in the nuclear volume analysed. Bar 10 μm. (**B**) The graphs illustrate the quantification of the nuclear volume and ellipticity (prolate) from cells in (A). (**C**) Schematic representation of different technical approaches used to determine the nuclear stiffness and viscoelasticity of isolated nuclei. The regions of interest analysed in each technique (the top region for AFM (Atomic Force Microscopy), and bottom form QCM-D) are indicated. (**D**) Isolated nuclei from Jurkat or primary CD4^+^ T-cells cultured in suspension and on VCAM1 were sedimented onto poly-Lysine coated slides, fixed with formaldehyde and analysed by AFM. Values from nine different points of each nucleus were taken. (**E**) Nuclei isolated as in (C) were attached to a quartz chip coated in poly-Lysine. The viscoelastic properties of the layer at confluence were calculated using the Voigt–Kelvin approximation. ** *P* < 0.01; ***P < 0.001.

Osmotic pressure affects the partition of water in cells and within cellular compartments and chromatin conformation responds to hypotonic and hypertonic conditions altering nuclear size, volume and geometry (30,31). We determined that Jurkat cells cultured on VCAM1 were less sensitive to nuclear changes in volume and ellipticity under osmotic shock, compared to control cells (Figure 6A and B, and Supplementary Figure S7). To follow this up and define the contribution of chromatin conformation to physical properties of the nucleus, we isolated nuclei from cells grown in different conditions, and analysed the nuclear stiffness and viscoelasticity using additional complementary approaches (Figure 6C). Isolated nuclei from Jurkat cells or primary CD4^+^ T-cells cultured in suspension, or on VCAM1 were sedimented onto poly-Lysine coated slides and their properties analysed by AFM. This revealed that nuclei from cells cultured on VCAM1 had greater apparent stiffness compared to control nuclei (Figure 6D). To confirm alterations in the physical properties of the nucleus, and to determine the role of G9a activity, we used a quartz crystal microbalance with dissipation (QCM-D) (32) to analyse changes in the mass and viscoelastic properties of isolated nuclei. Results showed a different viscoelasticity of isolated nuclei from Jurkat cells in response to cell adhesion *via* α4β1 compared to control cells, or cells cultured on ICAM1. Moreover, the effect induced by α4β1 adhesion could be inhibited partially with chaetocin, and completely with BIX01294 (Figure 6E). Our results indicate that epigenetic changes alter the nucleus of T-cells and its physical properties (stiffness and viscoelasticity).

### G9a activity influences T-cell migration

**Figure 7.**
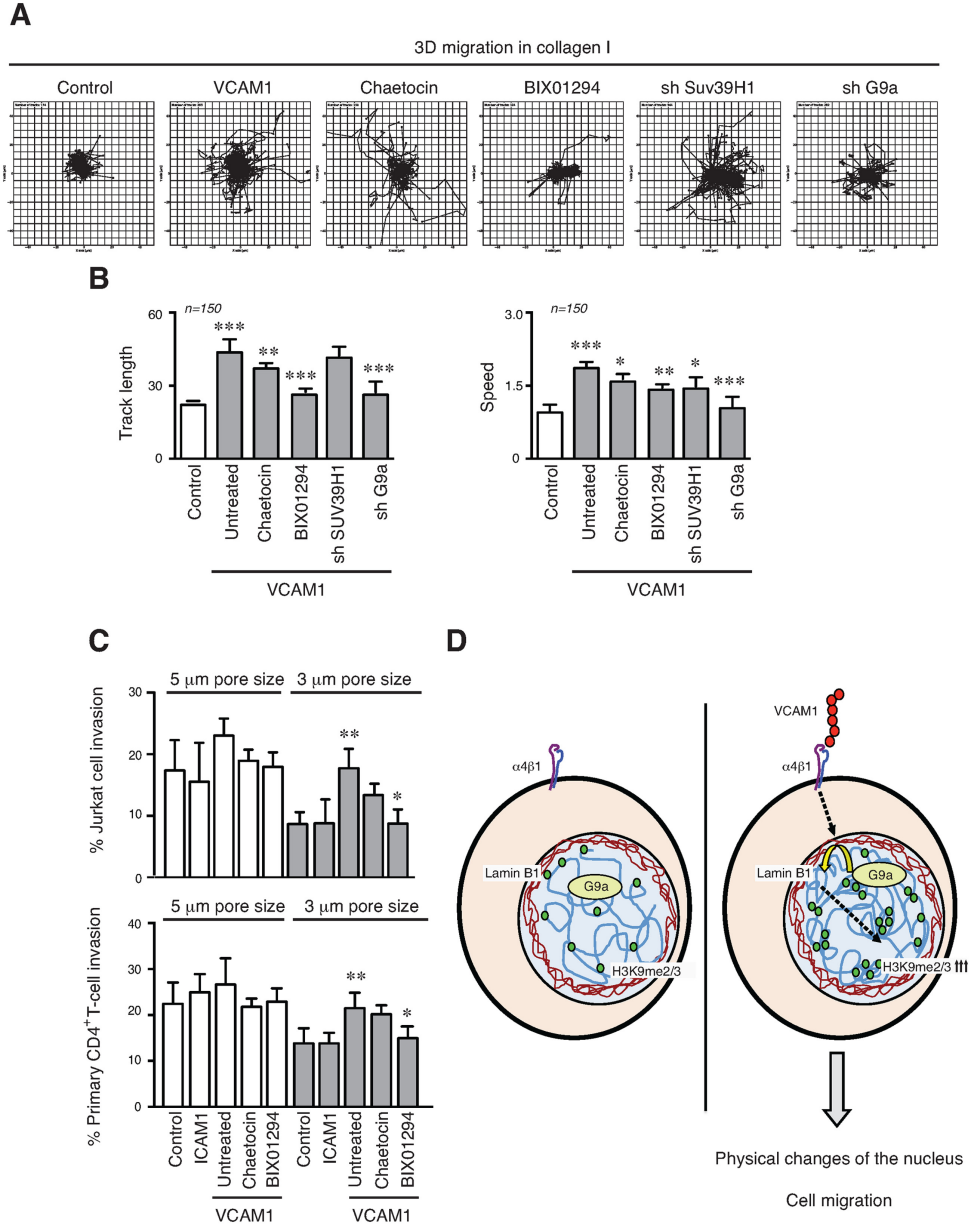
Epigenetic changes enhance the T-cell migration. (**A**) Jurkat cells were cultured for 24 h on VCAM1 in the presence or not of inhibitors. Depleted cells for suv39H and G9a were analysed. Then cells were labelled, collected and mixed in a 3D collagen matrix (1.7 mg/ml). Panels show results of cell trajectory plots. (**B**) Track length and velocities of migrating cells in collagen gel were quantified. (**C**) Jurkat cells (upper panel) or primary CD4^+^ T-cells (lower panel) were cultured on VCAM1 for 24 h in the presence or not of chaetocin or BIX01294. Then, cells were collected and migration quantified across Transwell filters (3- or 5-μm pore sizes). The cell migration was induced with 100 ng/ml CXCL12. Data are expressed as a percentage of input cells. (**D**) Schematic representation showing how in T-cells α4β1 integrin, upon adhesion to its ligand VCAM1, induces G9a recruitment at the nuclear envelope, resulting in upregulation of H3K9me2/3 levels. This leads changes in the chromatin structure, which contribute to the physical properties of the nucleus. These epigenetic changes induced by the integrin promote cell migration even through narrow spaces where high nuclear plasticity is required. *P < 0.05; **P < 0.01; ***P < 0.001.

We preincubated Jurkat or primary CD4^+^ T-cells with HMT inhibitors, and tracked their movement on VCAM1. Our results showed that HMT inhibitors reduced cell migration (Supplementary Figure S8). Then, we analysed how epigenetic changes induced by α4β1 affect lymphocyte migration in 3D collagen gels. Labelled Jurkat cells cultured in suspension or on VCAM1 in the presence or not of chaetocin and BIX01294, were cultured in 3D collagen gels and their movement was tracked. Migration velocity, displacement and distance were all increased in those cells cultured on VCAM1 for 24 h prior to the analysis compared to control cells in suspension, or treated with G9a inhibitor (Figure 7A and B). To confirm how HMT activity and epigenetic changes could affect functional T-cell movement, we used transwell filters with two different size pores, where the smallest requires higher cell deformability to enable migration (33). Whilst no significant differences were observed using the larger pores; Jurkat or primary CD4^+^ T-cells (Figure 7C) cultured on VCAM1 migrated more than control cells through the more restrictive transwell pores and this effect was sensitive to G9a inhibition.

These results demonstrated how HMT activity and epigenetic alterations induced by α4β1 promote changes in the physical properties of the nucleus, which are important in the control T-cell migration (Figure 7D). This effect might be highly relevant when cells are migrating within specific and restrictive conditions, which may be commonly found in tissue matrices.

## DISCUSSION

Regulation of T-cell migration and traffic through lymphoid organs is critical for lymphocyte homeostasis, inflammation and immune response. The role of integrins and other cytoskeletal proteins has been well documented, but the nuclear mechanisms mediating cell migration in lymphocytes remain unclear. In this study we show, for the first time, that cell adhesion *via* α4β1 integrin triggers G9a localisation and activity, which mediates specific epigenetic changes and affects physical properties within the nucleus that are required for cell migration.

The integrin α4β1 controls lymphocyte adhesion within the vasculature to endothelial cells and extravasation from the circulation (13,34). α4β1 regulates leukocyte recruitment into an inflamed tissue in several inflammatory diseases (35). It has been reported that melanoma cells, which also express α4β1 (36), present heterochromatin markers (H3K9me3, H4K20me1) during their migration (19). We have determined that lymphocyte adhesion through α4β1 promotes increased levels of H3K9me2/3 methylation, but not other epigenetic alterations (e.g. H4K20me3). Modest changes in global H3K9me3 levels, similar to those described in this study, are sufficient to induce cell responses such as proliferation, survival or migration (10,21,37,38). SUV39H1 is the principal HMT involved in H3K9 trimethylation; while G9a is mainly involved in the transfer of the first and second methyl residues and may add the third one in some conditions (39–41). G9a, which forms homo and heteromeric complexes with its partner GLP, is crucial during early embryo development (42,43). Both HMTs are required for epigenetic changes induced by the integrin β1 in response to matrix stiffness (21), and we have determined here that the methylation of H3K9 induced by α4β1 integrin is a major function of G9a with a partial contribution of SUV39H1. G9a controls CD4^+^ T-cell gene expression and differentiation through mechanisms both dependent and independent of its enzymatic activity (24,25). We demonstrate here that α4β1 adhesion requires G9a activity for H3K9 methylation; and this is independent of any specific cytokine production or process of Th2 differentiation. Nonetheless, we cannot rule out the possibility that specific cytokine stimulation would enhance the effects induced by α4β1, but this requires resolving in a future study.

Significantly, α4β1 adhesion organizes the disposition of diverse organelles in the cell body, and these are reported to control lymphocyte migration (44). In this study, we extend this concept to show that α4β1 integrin signalling also controls the nucleus as the biggest organelle in the cell body. Cell receptors transmit mechanical forces into the nucleus, affecting its physical properties and cell motility (45). Recently, it has been described how isolated nuclei respond to mechanical forces altering their stiffness, however, the contribution of chromatin remains unclear (46,47). Here, we determine by AFM that the nuclei from cells cultured on VCAM1 exhibited an apparent increase in stiffness, whereas with QCM-D the results showed a more elastic response. These techniques are complementary and measure different properties and nuclear areas (34), and our results clearly show that epigenetic changes alter the nucleus of T-cells and its physical properties (stiffness and viscoelasticity). The nucleus presents many different structures and compartments that contribute to nuclear viscoelastic properties (48); and is connected with cytoskeleton, helping the cells to elongate, spread or migrate (49). The chromatin compaction and altered physical properties of the nucleus within T-cell adhesion could be more deformed by cytoskeletal forces promoting the migration of T-cells.

Multiple nuclear envelope proteins interact with chromatin in LADs (lamina-associated domains) (50). T-cells express lamin A, (the major nuclear envelope protein in most cell types), only under specific conditions (51); however, we did not observe a significant level of this protein or change in lamin B1 expression during cell adhesion. Lamin-chromatin interactions are highly dynamic and SUV39H1 (28) or G9a activity (27) could trigger chromatin anchoring to the nuclear envelope. Furthermore, lamin B1 associates G9a/GLP activity and H3K9me2/3 regions in large organised chromatin lysine modification blocks (LOCKs) during the processes of stem cell renewal, hematopoiesis and cancer progression (52,53). Interestingly, we found that G9a was associated with lamin B1 when cells are cultured on VCAM1, indicating a possible link between chromatin and the nuclear envelope. The specific changes of H3K9 methylation described by us and others (10) might influence the nuclear envelope, suggesting new potential roles of these proteins during immune responses and lymphocyte migration.

Finally, epigenetic mechanisms could provide new pharmacological targets for multiple human pathologies, including cancer, neurological dysfunctions, inflammatory and immune disorders (5,6). Inhibitors against epigenetic machinery induce defects in cell growth, differentiation and progression in tumour cells (54). Although integrins may not be required for lymphocyte migration across 3D matrix (55), α4β1 integrin could influence homing of T-cells into sites of inflammation or into constrictive conditions where VCAM1 is present or upregulated. For example, these include asthma, multiple sclerosis, rheumatoid arthritis and blood cancers (35). This is significant because in these contexts, α4β1 could control epigenetic changes and nuclear deformability, thereby facilitating the ability of these cells to migrate through tissues. Our findings illustrate, for first time, how HMT inhibitors affect lymphocyte migration, and open new questions about how epigenetic inhibitors could block cell migration.

In summary, we have found that T-cell adhesion through α4β1 integrin affects the levels of H3K9me3, the global chromatin structure and the physical properties of the nucleus, including volume, shape and viscoelasticity. Remarkably, the HMT G9a is the major contributor in the methylation of H3K9 mediated by α4β1 adhesion, and its absence or inhibition leads to the recovery of normal nuclear properties. Furthermore, blocking these epigenetic changes inhibits lymphocyte migration, suggesting that depending on physiological context, this integrin-mediated interaction regulates cell migration by alterations in chromatin and in the structure and physical properties of the nucleus.

## Supplementary Material

SUPPLEMENTARY DATA
