# Supplementary material for: Integrin α4β1 controls G9a activity that regulates epigenetic changes and nuclear properties required for lymphocyte migration
Source: Nucleic Acids Res. 2015 Dec 10;44(7):3031–44. doi: 10.1093/nar/gkv1348 (PMC4838336; doi:10.1093/nar/gkv1348)
Supplement: SUPPLEMENTARY DATA [file supp_44_7_3031__index.html]

Integrin α4β1 controls G9a activity that regulates epigenetic changes and nuclear properties required for lymphocyte migration — Integrin α4β1 controls G9a activity that regulates epigenetic changes and nuclear properties required for lymphocyte migration — SUPPLEMENTARY DATA 

# Integrin α4β1 controls G9a activity that regulates epigenetic changes and nuclear properties required for lymphocyte migration

## SUPPLEMENTARY DATA

- SUPPLEMENTARY DATA
