# Supplementary material for: Integrin α4β1 controls G9a activity that regulates epigenetic changes and nuclear properties required for lymphocyte migration
Source: Nucleic Acids Res. 2015 Dec 10;44(7):3031–44. doi: 10.1093/nar/gkv1348 (PMC4838336; doi:10.1093/nar/gkv1348)
Supplement: SUPPLEMENTARY DATA [file supp_gkv1348_nar-02456-m-2015-File009.pdf]

Supplementary Data

Integrin α4β1 controls G9a activity that regulates epigenetic changes and nuclear properties required for lymphocyte migration.

X. Zhang, P.C. Cook, E. Zindy, C.J. Williams, T.A. Jowitt, C. Streuli, A.S. MacDonald, J. Redondo-Munoz

This supporting information contains:

Supplementary Materials and Methods

Supplementary Table S1.

Supplementary Figures.

Supplementary Materials and Methods

Immunofluorescence. Jurkat cells were cultured for 24 h on poly-Lysine, ICAM1 and VCAM1 coated coverslips. Cells were fixed in 4% formaldehyde (10 min), permeabilised with 0.5% Tx-100 PBS (5 min), blocked in 10% horse serum and incubated with appropriated primary antibodies for 1 h at RT. After several washes, samples with incubated with secondary antibodies. Samples were mounted in Dako and imaged with a Leica TCS SP5 confocal microscope. For H3K9me2/3 and H4K20me3 staining, after fixation samples were incubated in 100 mM sodium citrate preheated at 95ºC for 20 min prior cell permeabilisation.

Quantification of the G9a localisation at the nuclear envelope was performed on 2-D confocal images (horizontal plane passing through the middle of the cells), using CellProfiler (available from CellProfiler Project website http://www.cellprofiler.org). An image-processing pipeline was devised to measure the mean pixel brightness of the G9A antibody staining in both the nucleic membrane annulus and remaining central region of the nuclei. Nuclei segmented in the Hoechst channel were eroded by 10 pixels to produce the central regions, and these were then dilated in the G9a channel up to the nuclei edge, or by at most 20 pixels, thereby defining the total G9a region. Subtracting the central nucleic region from the total region gives us the nucleic membrane annulus. To quantify H3K9me2/3 staining, the mean intensity of a determined cell population was measured in the anti-H3K9me2/3 channel

after background subtraction. H3K9me2/3 and H4K20me3 foci counting analysis were performed manually using ImageJ.

RT-qPCR. Total RNAs were isolated by using RNAqueous-Micro Total RNA Isolation Kit (Life Technologies), according to the manufacturer's instructions. RNA were converted into cDNA by TaqMan Reverse Transcription Reagents (Life Technologies), qPCR was performed using the StepOnePlus System (Life technologies) and data were normalized to expression of two reference genes, ACTB and GAPDH. Gene expression is relative to that of the control samples. Values shown are the mean ± standard deviation of three independent experiments. Primer sequences are shown as Supplemental table 1.

Cellular treatments. Integrin activation was performed with CXCL12 (150 ng/ml), Mn2+ (1 mM), Mg2+ (1 mM), 12G10 (5 μg/ml) and 9EG7 (5 μg/ml) for 24 h at 37°C. For activation via CD3 crosslinking, anti-CD3 antibody (2 μg/ml) was co-immobilized with VCAM-1 and used as adhesion substrate. For IL-4 stimulation, cells were incubated in the presence or not of IL-4 (20 ng/ml).

Production of lentivirus Short hairpin RNA sequences encoding sequences targeting G9a (GGACCTTCATCTGCGAGTATG); SUV39H1 (AGTCGAGTACCTGTGCGATTA) and lamin B1 (CCAGGGAAGAACTGATGGAAAT) were purchased from Sigma-Aldrich and inserted in the pVLVTHM vector. All recombinant lentiviruses were achieved using the pVenus lentiviral transfer vector, (pLVTHM) and generated by transient transfection of 293T cells using Pei according to manufacturer's protocol. Briefly, subconfluent HEK293T cells were co-transfected with the

pVLVTHM vector and the packaging vectors pPsPax2, pMD2G. After 24 h, the medium was replaced with fresh medium 10 mM sodium butyrate and media changed after 6h. 48 h later virus containing supernatants were harvested and filtered through 0.45 µm pore-sized membranes. Infection was performed by adding the lentiviral containing media to Jurkat cells at $1x10^6$ cells/ml, with 10 µg/ml Polybrene (Millipore; Carlsbad, CA). The media was changed after 24 hours, and cells were passaged over two weeks. Stably infected cells were sorted by FACS based upon GFP fluorescence.

QCM-D. Silicon dioxide sensors were cleaned by sonicating in 2% SDS followed by rinses in water and 100% methanol before drying under a flow of nitrogen. The sensors were then incubated for 20 minutes in a UV/Ozone cleaner (BioForce Nano ProCleaner plus, Bioforce Nanosciences). Sensors were mounted into a E1 QCM-D instrument (Biolin Scientific) and equilibrated in 10 mM Tris-HCl pH 7.4 with 150 mM NaCl, 1 mM Ca2+ and 1 mM Mg2+ (running buffer). The fundamental resonance frequency of the silicon dioxide coated sensors (QSX303, Biolin Scientific) was established along with the corresponding overtones 3, 5, 7, 9 and 11 corresponding to resonance frequencies of 15, 25, 35, 45 and 55 MHz respectively and constant flow of 50 µl/min at 200C. In all cases starting frequencies were offset to zero prior to the experiment. Nuclei from cells cultured in suspension, on ICAM1 or VCAM1 (with and without inhibitors) were isolated and their association to the surface was achieved by coating the sensors in poly-L-lysine (Sigma-Aldrich) by injecting 500 µl of 0.1 mg/ml solution in running buffer. This was followed by a 500 µl injection of a 1/10 dilution of nuclei giving a concentration of 10^5

nuclei/ml. The viscoelastic properties of the surface following nuclei absorption were calculated using the overtones 5, 7 and 9 which correspond to a viscoelastic penetration depth of the surface of ~100 nm using the Kevin-Voight model within the QCM-D application software (Biolin Scientific).

MNase digestion. To analyse the nucleosomal profile, cells of interest were collected, lysed in lysis buffer (10 mM Tris [pH 7.5], 10 mM NaCl, 2 mM MgCl2, 0.5% NP-40, 1 mM CaCl2) with protease inhibitors (Roche) and incubated for 15 min at 4°C. and MNase digestion performed with 100U of MNase (New England Biolabs) at 37°C. Reactions were stopped adding EDTA, incubated for 10 min at 37°C with RNaseA (Life Technologies) and then incubated overnight at 65°C with 0.01% SDS and proteinase K (New England Biolabs). Digested DNA was washed with phenol/chloroform/isoamyl alcohol (25:24:1) and precipitated by 3 M sodium acetate and 2.5 volumes of ethanol. DNA pellet was dissolved in water and nucleosomal releasing resolved in 2% agarose gel.

Cell adhesion assay. Jurkat cells were treat with Chaetocin (0.5 µM) or BIX01294 (1 *µ*M) for 30 min prior adding into 96-well plates coated with ICAM1 and VCAM1 (both at 5 *µ*g/ml). The wells were gently washed with PBS twice and the adherent cells were fixed with formaldehyde (4%) in PBS. Three areas/well were selected randomly and the adherent cells were counted under a microscope.

Transwell cell migration assay. We used transwell plate inserts (Corning Costar, 6.5 mm diameter, 3 µm or 5 µm pore sizes). 600 µL of serum free medium and 100 µL of a cell suspension (2×10^5 cells/well) were added to

the lower chamber and upper chamber, respectively. CXCL12 (100 ng/mL) was added to the lower chamber and the chambers were incubated in a CO_2 incubator at 37 °C for 3 h. Migrated cells were collected and counted using a hemacytometer.

Cell migration assay. We coated 96-well plates with poly-Lysine (5 µg/ml) or VCAM1 (2.5 µg/ml). Jurkat cells pretreated or not with chaetocin (0.5 µM) or BIX01294 (1 µM) for 30 min were labelled with Hoechst 33342 and then plated onto wells at 37°C and 5% CO_2. Time-lapse images were acquired on an AS-MDW live-cell imaging system (Leica). Point visiting was used to allow multiple positions to be imaged within the same time course, and cells were maintained at 37°C and 5% CO2. Images were collected every 2 min over 30 min (sparsely plated cells). Cell migration was tracked using Imaris. Cell migration through a 3D collagen matrix was assayed according to the manufacturer's instructions (Ibidi; Martinsried, Germany). Jurkat cells were cultured under different conditions for 24 h. Then, cells were preincubated with CFSE (cells in suspension or treated with inhibitors) or cell tracer Far Red (cells onto VCAM1) both at 5 µM for 10 min, washed, mixed and embebed in a 3D collagen matrix. Random cell migration was analysed from images taken every 5 min for 3 h. Images were acquired on a Nikon TE2000 PFS microscope using a 20x/ 0.5 Plan Fluor objective and the Sedat filter set Chroma (89000). The images were collected using a Cascade II EMCCD camera (Photometrics) with a Z optical spacing of 0.2 µm.

Immunoprecipitation and Immunoblotting. Cells were lysed in RIPA buffer for 30 min at 4C and then sonicated using a Microson XL2000 (Misonix;

Farmingdale, NY). After preclearing with protein G-Agarose beads (Pierce), supernatants were incubated with antibodies followed by coupling to protein G- or protein A-Sepharose. Proteins were resolved by SDS-PAGE. For total lysates, proteins were extracted using SDS-loading buffer and sonication before boilling. Proteins were resolved in 12.5% polyacrylamide gels. Gels were transferred to nitrocellulose membranes then blocked in 5% low fat milk in TBS-Tween (0.5%) for 1 hour at room temperature. Membranes were incubated with primary antibodies in 5% low fat milk in TBS-tween (0.5%) at the appropriate dilution at 4°C overnight. Membranes were washed in TBS-Tween (0.5%) and incubated with appropriated IRDye secondary antibodies (Li-cor Biosciences; Lincoln NE) in 5% low fat milk in TBS-Tween (0.5%) for 1 hour at room temperature. Protein signal was analysed by Odyssey (Li-cor).

Osmotic stress analysis. Jurkat cells were cultured on coated plates for 24 h. Then, medium was replaced with hypotonic (normal medium diluted 1:5 with water) or hypertonic (medium with 0.4 M NaCl) medium and incubated for 5 min before fixation with 4% formaldehyde (10 min). Cells were permeabilized with 0.5% Tx-100 PBS (5 min), blocked and stained by Hoechst 33342. Nuclear shape and volume were analysed by confocal microscopy using an inverted confocal (TCS SP5 AOBS; Leica) with 40.0x, 1.25 NA HCX Plan Apochromat oil or 63×, 1.4 NA HCX Plan Apochromat oil objectives and LAS AF acquisition software (Leica). Images were analyzed with ImageJ (NIH; Bethesda, MD), and Imaris TM software (Bitplane).

Supplementary Table S1

oligo name	Sequence 5'- 3'
Hu-IL-4-F	CACCGAGTTGACCGTAACAG
Hu-IL-4-R	GCCCTGCAGAAGGTTTCC
Hu-IL-5-F	CACTGAAGAAATCTTTCAGGGAAT
Hu-IL-5-R	CCGTCTTTCTTCTCCACACTTT
Hu-IL-6-F	GATGAGTACAAAAGTCCTGATCCA
Hu-IL-6-R	CTGCAGCCACTGGTTCTGT
Hu-IL-10-F	GAAATGTGTCAGGAGCGATG
Hu-IL-10-R	ATCCAGGAGCTGTCCCTCA
Hu-IL-13-F	AGCCCTCAGGGAGCTCAT
Hu-IL-13-R	TGATGCTCCATACCATGCTG
EHMT2-F	TGGAGATCTGCCATGTGCT
EHMT2-R	CTGCTGTTTGTCCACTGCAT
SUV39H1-F	GTCATGGAGTACGTGGGAGAG
SUV39H1-R	CCTGACGGTCGTAGATCTGG
hGAPDH-F	CTGGGCTACACTGAGCACC
hGAPDH-R	AAGTGGTCGTTGAGGGCAATG
hActb-F	GCCGCCAGCTCACCAT
hActb-R	AATCCTTCTGACCCATGCCC

Supplementary figure legends

Supplementary Figure S1 related to Figure 1. Disposition of H4K20me3 is not affected upon cell adhesion *via* integrins. (**A**) Jurkat cells were cultured on poly-Lysine, ICAM1 or VCAM1 (all at 5 μg/ml). After 24 h cells were fixed and stained with Hoechst (DNA, blue), anti-lamin B1 (nuclear envelope, red) and anti-H4K20me3 (heterochromatin, green). Bar 10 µm. (**B**) Graph shows the fluorescence intensity quantified from (A). (**C**) Number of heterochromatin H4K20me3 foci were counted in cells from (A).

Supplementary Figure S2 related to Figure 2. Integrin activation is not enough to induces epigenetic changes. (**A**) Primary $CD4^+$ T-cells were preincubated with blocking antibodies against -α4 (HP2/1) and -β1 (mab13) integrin subunits, 30 min prior cell culture onto VCAM1. After 24 h, protein levels were analysed by Western blotting. (**B**) Jurkat or primary $CD4^+$ T-cells were cultured in suspension, on VCA1, FN-H50, FN-H120 (all at 5 μg/ml) for 24 h and lysates analysed by Western blotting. (**C**) Jurkat cells were cultured for 24 h in the presence of β1-activator antibodies or divalent cations (Mn^{2+}, Mg^{2+}, or Ca^{2+}). Epigenetic changes were analysed by western blot.

Supplementary Figure S3 related to Figure 3. Effects of HMT inhibition on cell adhesion and epigenetic changes induced by α4β1. (**A**) Jurkat cells cultured on VCAM1 in the presence or not of actinomycin (1 μg/ml), chaetocin (500 nM) or BIX01294 (1 μM) were fixed and stained with Hoechst (blue), and anti-H3K9me2/3 (red). Bar 10 µm. H3K9me2/3 fluorescence intensity, or number of heterochromatin foci per nucleus were quantified of cells. (**B**) Jurkat cells were pretreated with chaetocin or BIX01294 and

cultured on plates coated with ICAM1 or VCAM1 (both at 2.5 μg/ml). Cell adhesion was determined after 30 min. (**C**) Jurkat cells were infected with specific shRNAs for G9a, SUV39H1 and control. After sorting, knockdown expression was confirmed by western blotting. (**D**) Depleted cell lines were cultured in suspension or on VCAM1 and levels of H3K9me2/3 analysed by western blotting.

Supplementary Figure S4 related to Figure 4. Epigenetic changes induced by cell adhesion are independent of Th2 differentiation. (**A**) Jurkat cells were cultured onto VCAM1 in the presence or not of IL-4 (500 units/ml) for 24 h. Cell lysates were analysed by western blot. (**B**) qPCR analysis of the expression of several IL genes that play a role in Th2 differentiation and response in Jurkat cells cultured onto different ligands.

Supplementary Figure S5 related to Figure 4. Depletion of lamin B1 affects the epigenetic changes induced by α4β1 adhesion. (**A**) Jurkat cells were infected with specific shRNAs for lamin B1. After sorting, knockdown expression was confirmed by Western blotting. (**B**) *In vitro* H3K9 HMT enzymatic assay using nuclear fractions from lamin B1-depleted cells in suspension or onto VCAM1.

Supplementary Figure S6 related to Figure 5. G9a inhibitor affects chromatin structure of cells plated on VCAM1. (**A**) are more sensitive to DNA digestion. Jurkat cells were cultured on VCAM1 in the presence or absence of different inhibitors for 24 h. Then, DNA was digested by DNaseI before fixation and nuclei were stained with Hoechst-33342 and their volume and area analysed. Bar 10 μm. (**B**) Jurkat cells were cultured on VCAM1 in

the presence or absence of different inhibitors for 24 h. Then, their nuclei were digested with micrococcal nuclease. DNA fragments were purified, resolved in agarose gel and the mononucleosomes (1n), dinuclueosomes (2n) and trinucleosomes (3n) quantified.

Supplementary Figure S7 related to Figure 6. 3D reconstruction of nuclei, according to their volume, from cells cultured under osmotic stress. Jurkat cells cultured on poly-Lysine or VCAM1 under osmotic stress conditions were fixed and stained with Hoechst. Reconstruction of nuclei was done from images and changes in ellipticity analysed. Bar 10 µm.

Supplementary Figure S8 related to Figure 7. HMT inhibitors affect migration in resting cells. (**A**) Cell trajectory plots of Jurkat cells pretreated with chaetocin or BIX01294 migrating onto VCAM1 (2.5 µg/ml) coated plates for 30 min. (**B**) Velocity (speed) and track length were quantified.

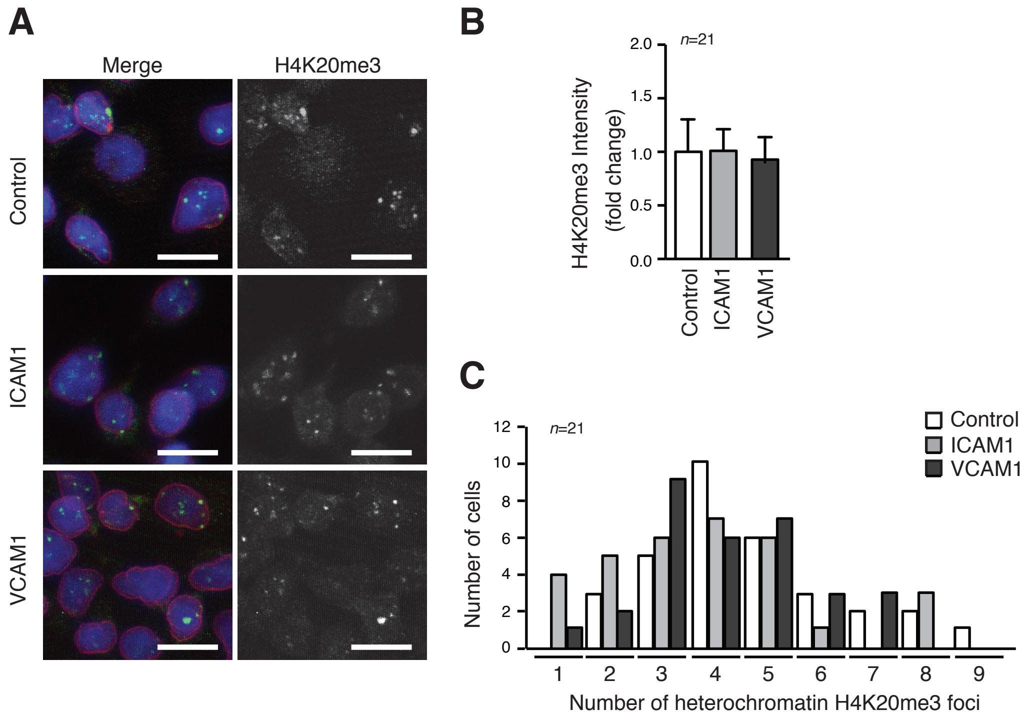

Supplementary Figure S1

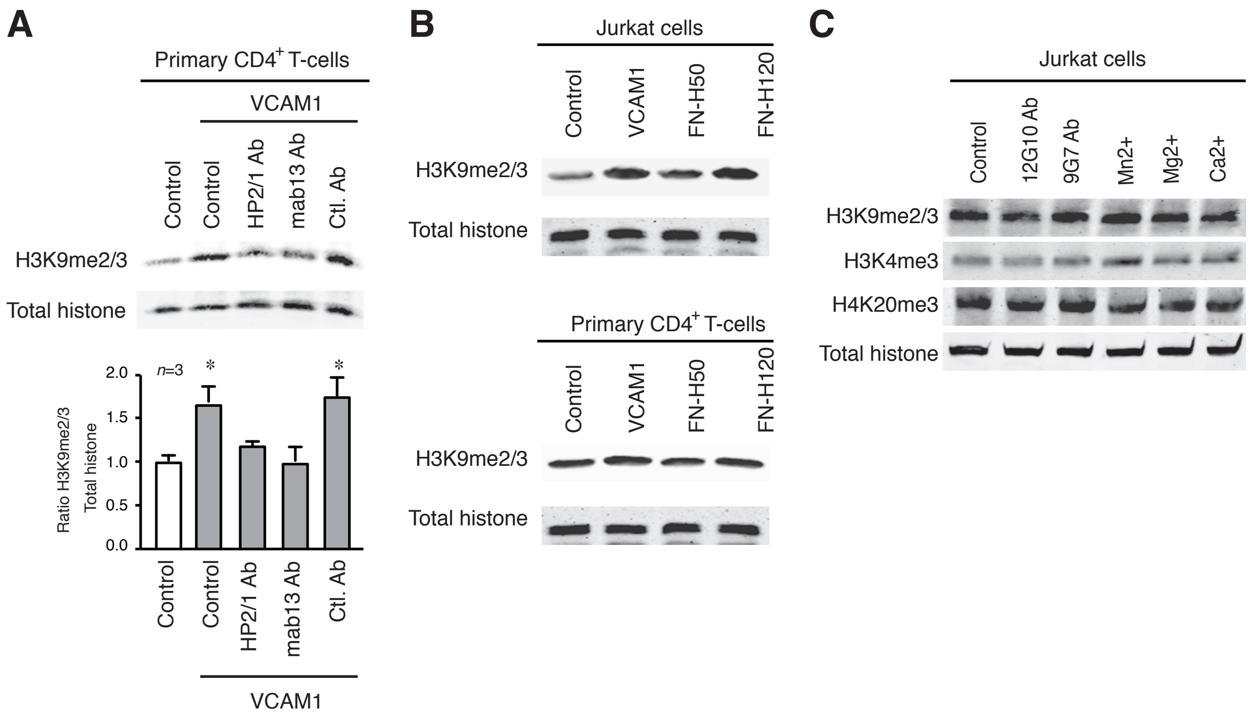

Supplementary Figure S2

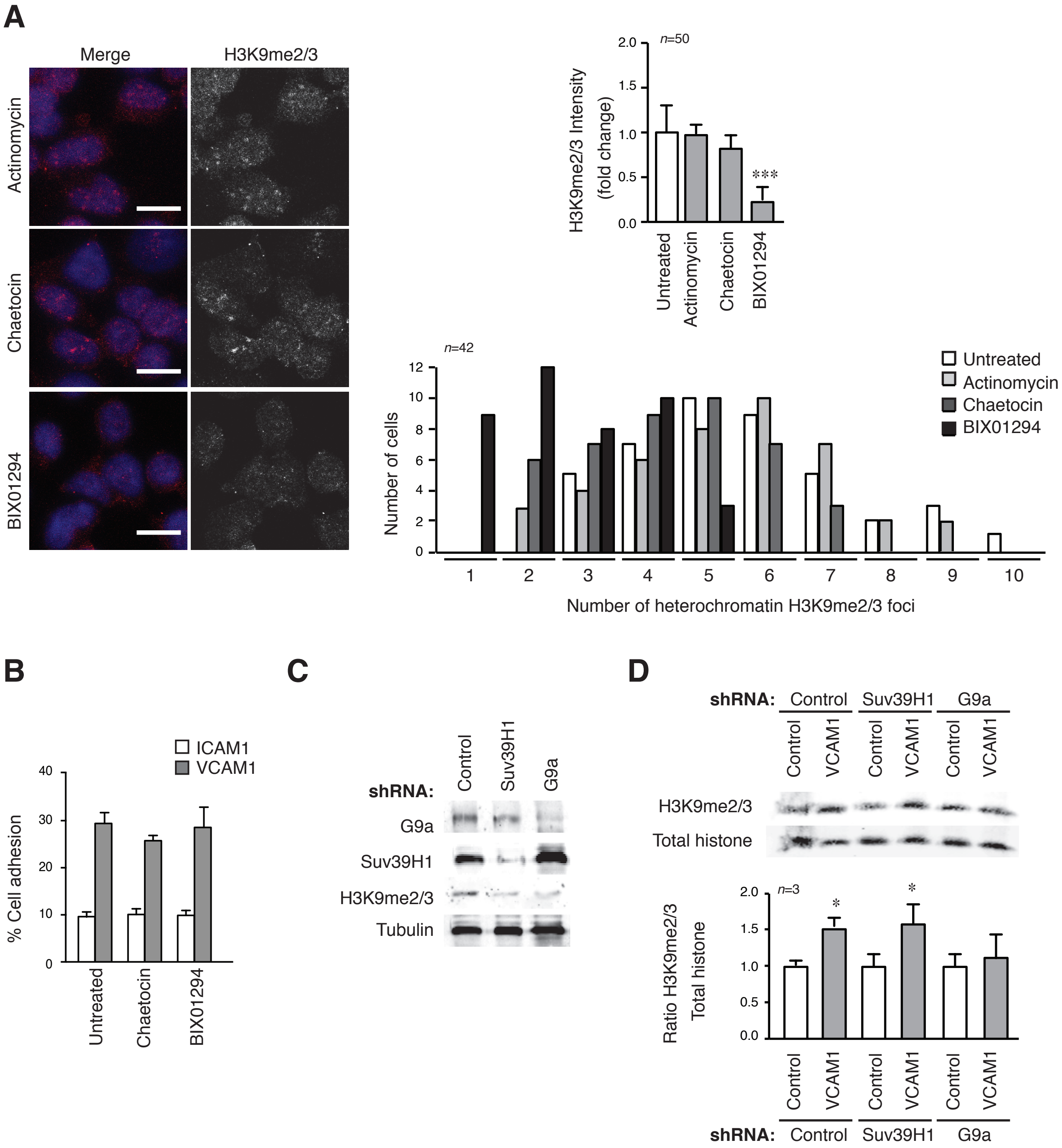

Supplementary Figure S3

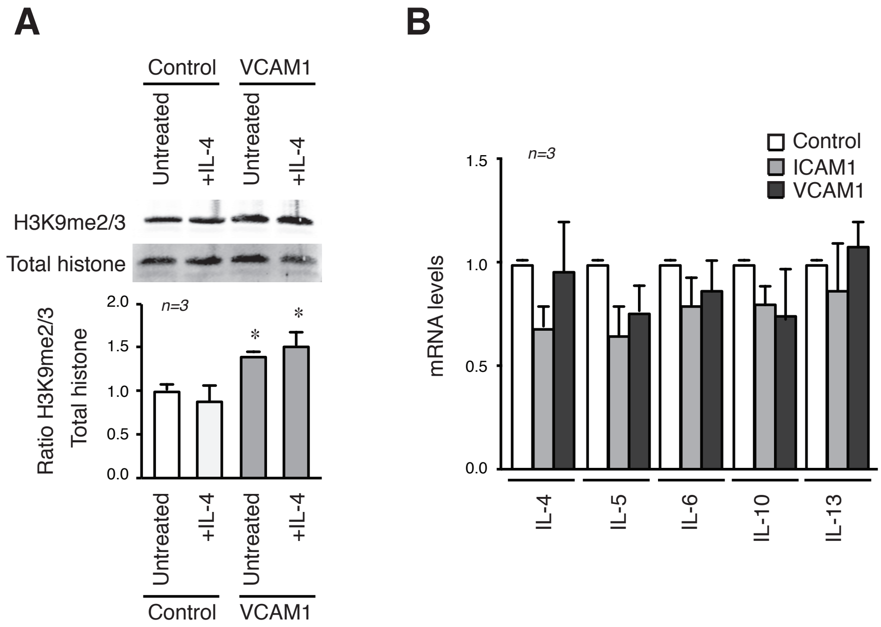

Supplementary Figure S4

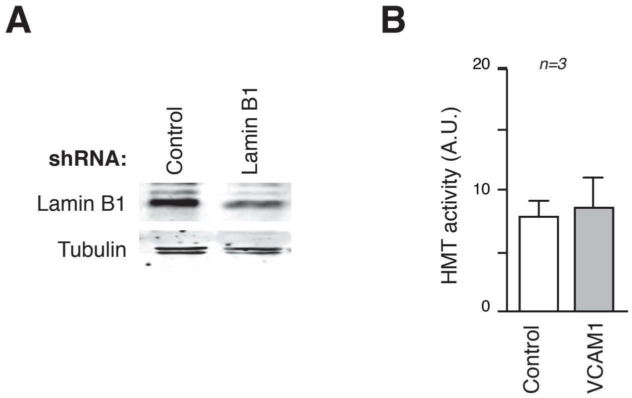

Supplementary Figure S5

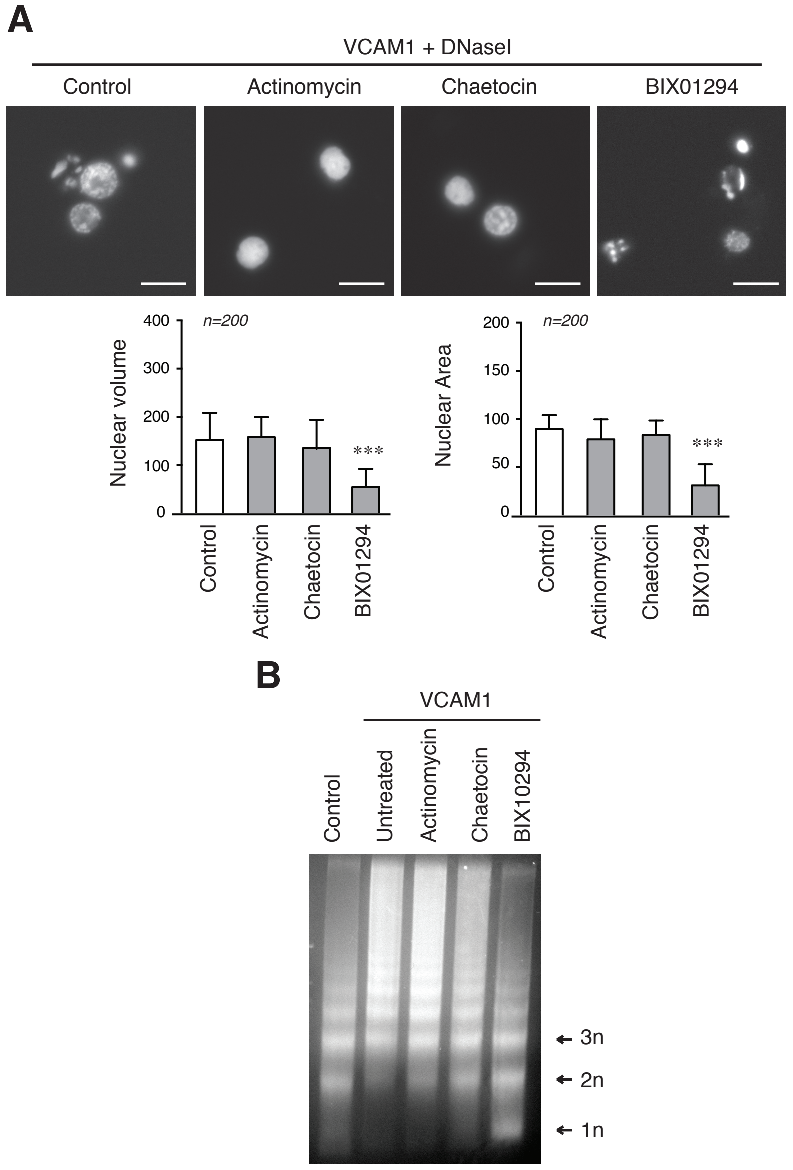

Supplementary Figure S6

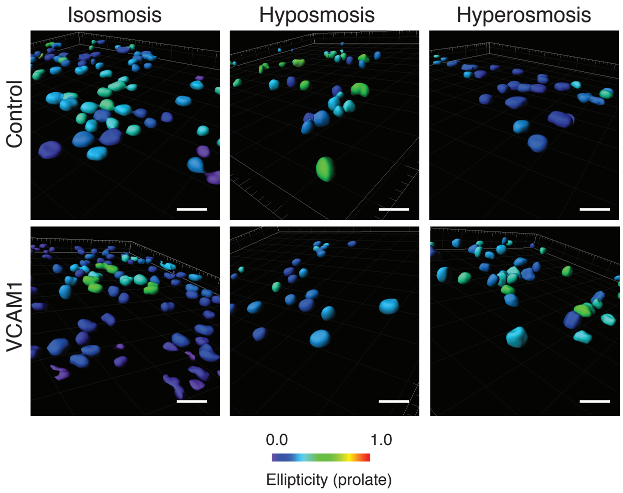

Supplementary Figure S7

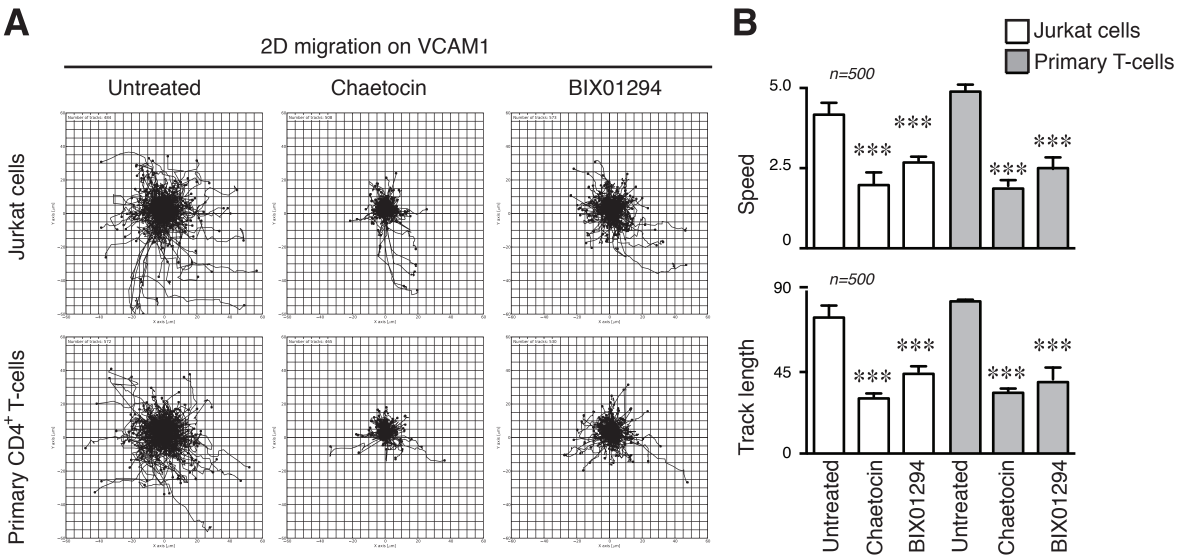

Supplementary Figure S8
